# Highly divergent lineage of narrow-headed vole from the Late Pleistocene Europe

**DOI:** 10.1038/s41598-019-53937-1

**Published:** 2019-11-28

**Authors:** Mateusz Baca, Danijela Popović, Anna Lemanik, Katarzyna Baca, Ivan Horáček, Adam Nadachowski

**Affiliations:** 1 Centre of New Technologies, University of Warsaw, Banacha 2c, 02-097 Warsaw, Poland; 2 Institute of Systematics and Evolution of Animals, Polish Academy of Sciences, Sławkowska 17, 31-016 Krakow, Poland; 3 Department of Zoology, Charles University, Viničná 7, 128 44 Prague, Czech Republic

**Keywords:** Phylogenetics, Biogeography

## Abstract

During the Late Pleistocene, narrow-headed voles (*Lasiopodomys gregalis*) inhabited Eurasia’s vast territories, frequently becoming the dominant small mammal species among steppe-tundra communities. We investigated the relationship between this species’ European and Asiatic populations by sequencing the mtDNA genomes of two extant specimens from Russia and 10 individuals from five Central European sites, dated to the post-LGM period. Phylogenetic analyses based on a large portion of mtDNA genomes highly supported the positioning of *L. gregalis* within *Arvicolinae*. The phylogeny based on mtDNA cytochrome *b* sequences revealed a deep divergence of European narrow-headed voles from Asiatic ones and their sister position against the extant *L. gregalis* and *L. raddei*. The divergence of the European lineage was estimated to a minimum 230 thousand years ago. This suggest, contrary to the current biogeographic hypotheses, that during the interglacial periods narrow-headed vole did not retreat from Europe but survived the unfavourable conditions within the refugial areas. Based on this result, we propose to establish a cryptic species status for the Late Pleistocene European narrow-headed vole and to name this taxon *Lasiopodomys anglicus*.

## Introduction

**Figure 1:**
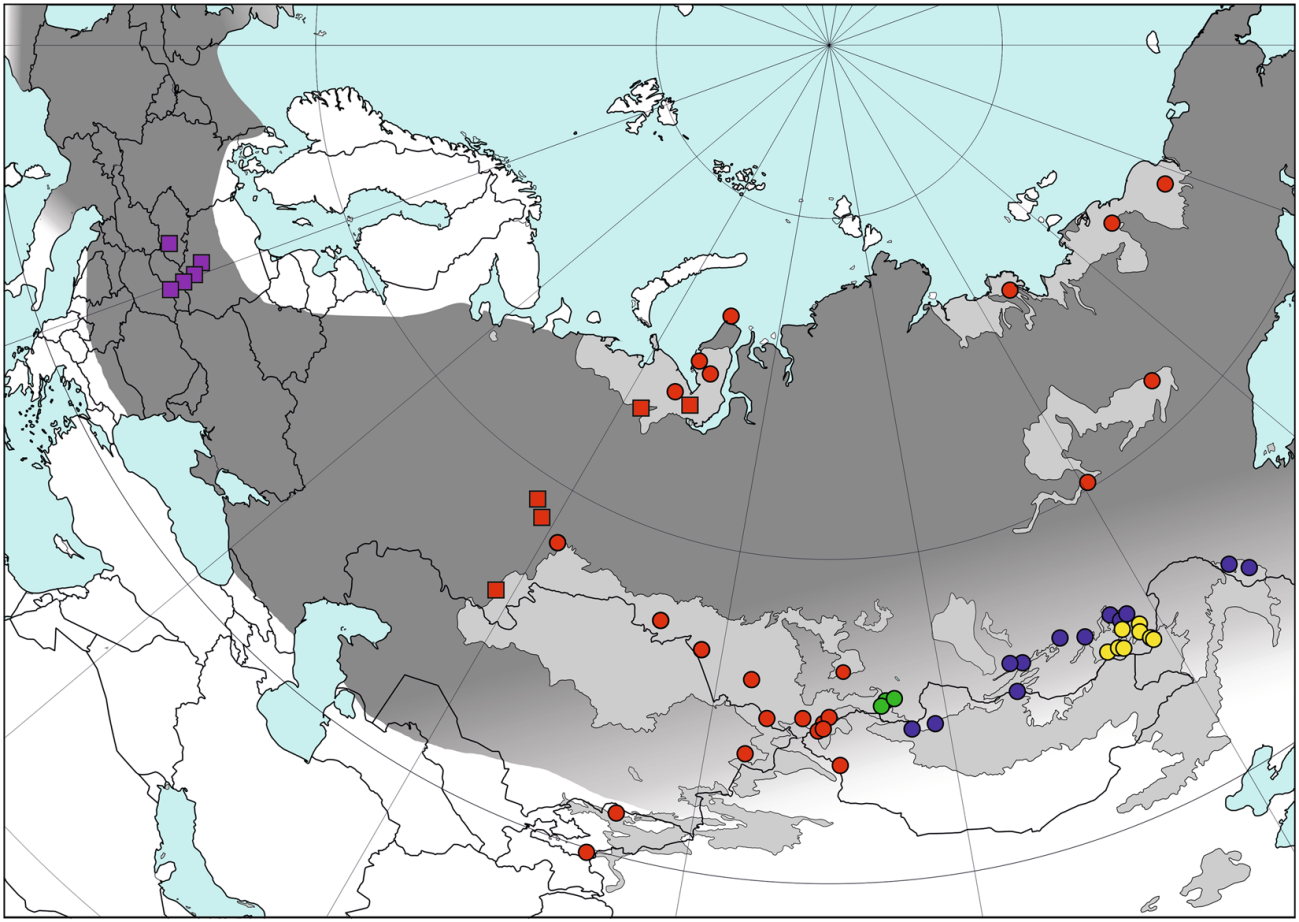
The Late Pleistocene and extant ranges of narrow-headed voles. The extant distribution (light gray) is given according to Shenbrot and Krasnov^1^ and the Late Pleistocene one (dark gray) is compiled from the literature. The southern range in Asia is marked tentatively. Circles denote sampling localities of extant narrow-headed voles from previous studies^16^ while squares indicate the Late Pleistocene sites. Color corresponds to the main mtDNA lineages of *L. gregalis* (red – A; blue – B; green – C) and *L. raddei* (D - yellow). Violet denote the newly recognized lineage E. The source map was made using public domain data from www.naturalearthdata.com. Range of the extant narrow-headed vole was downloaded from iucn.org.

Although the extant narrow-headed vole (*Lasiopodomys gregalis*) inhabits Asia’s vast territories, its range is disjunct into several areas of tundra in Arctic Russia from Northern Dvina River, Yamal Peninsula, to Kolyma River, forest-tundra in Central Yakutia and steppe and forest-steppe belt from Kama River East to Amur River, and from south Siberia, Tian Shan to interfluve of rivers Huang He and Yangtze in East China^1^ (Fig. 1).

During the Late Pleistocene, its habitat range was much larger, including nearly the whole Northern Eurasia. In Europe, it reached the Northern^2^ or even the Central Spain^3^, as well as the British Isles^4^. Its southern distribution has its limits in Northern^5^ or even Central Italy^6,7^, Serbia^8^, Northern Bulgaria^9^, Crimea^10^ and the area near Azov Sea in the European Russia^11^. Throughout the Late Pleistocene, the narrow-headed vole was either the dominant or subdominant small mammal species, within steppe-tundra environments of many regions in the European Plain, from southern France^12^ through Benelux Countries^13^, Central Europe^14^ to Ukraine and Russia^15^ (Fig. 1). The extinction of European populations and the fragmentation of the Asiatic habitat range probably occurred during the Holocene, with the expansion of forests^16^.

Among extant Asiatic populations, genetic variation is well recognised, comprising four main mtDNA lineages (A-D) with parapatric distributions. Lineage A covers most of the species range and is further subdivided into six sublineages (A1-A6) confined to specific geographic regions^16^. The main lineages’ divergence was estimated to occur between 0.75 Ma and 0.25 Ma years ago, with the most divergent Transbaikal lineage D being recently raised to the status of cryptic species *Lasiopodomys raddei*^17^. It was suggested that despite the Holocene’s range contraction, narrow-headed vole populations maintained a high genetic diversity^16^. This was supported by analyses of ancient DNA from Late Glacial and Holocene specimens from Middle and Northern Ural sites which showed increase of effective narrow-headed vole population size during the LGM (Last Glacial Maximum) and only slight decrease during the Late Glacial warming^18^. High morphological variation accompanies genetic variability. For example, vole populations from Polar Urals’ Eastern slopes, both subfossil and recent, exhibit high variability in terms of m1 length and morphotypes, comparable to those observed within the whole species range, but without any evident time trends nor continuity throughout the Late Pleistocene, Holocene until modern times^11^.

The relationship between Asiatic and European fossil populations has not been intensively studied. After taxonomic revision of Sutcliffe and Kowalski^4^ all authors accepted *Microtus gregalis* as a correct available name for the European narrow-headed voles. Only Rekovets^19^, on the basis of material from the LGM site Novgorod-Severskiy, classified Late Pleistocene narrow-headed voles from Ukraine as the separate subspecies *M. g. kriogenicus*, which went extinct at the beginning of the Holocene. Other studies showed that the high morphological variation of m1 teeth of Late Pleistocene European voles^20,21^ is within the range of variation of voles from North-eastern Europe and Western Siberia^11,22^. Differences between Late Pleistocene populations lie mainly in the proportion of different molar morphotypes (m1), although classification is sometimes difficult due to the presence of many intermediate forms. It was also shown that, in narrow-headed voles, high morphological similarity may be accompanied by high genetic differentiation^11^.

To better understand the relationship between European and Asiatic populations of narrow-headed voles we sequenced mtDNA genomes from 10 specimens excavated from Late Pleistocene sites in Central Europe (Fig. 1).

## Results

We *de novo* assembled mtDNA genomes of two present-day narrow-headed voles from the Southern Ural region, which resulted in 16,292 bp long sequences with the typical mammal gene order. One such sequence was subsequently used as a reference for mapping the reads obtained from the sequencing of Late Pleistocene samples. Ten samples from five sites yielded over 70% of the mtDNA genome sequences covered at least twice, which were used for phylogenetic analyses (Table 1). All samples exhibited excess of damage at the ends of the DNA molecules, typical of ancient DNA (Table 1).

**Table 1:** Samples used in the study and details of mtDNA sequencing.

Lab ID	Country	Site	Layer	Assigned age (years BP)^*^	Material	Reads	Reads mapped to mtDNA	Reads mapped to mtDNA without duplicates	mtDNA genome coverage (SD)	% of bases covered at least two times	Number of bases covered out of 9,090 bp	Deamination pattern**	
												3’end	5’end
MI068	Poland	Obłazowa cave (WE)	III	14,376	molar	665,460	5,657	1,014	5.96 (4.48)	86.64	7,811	0.22	0.23
MI069	Poland	Obłazowa cave (WE)	III	14,376	mandible	1,404,204	23,527	5,053	26.71 (16.85)	96.51	8,995	0.24	0.23
MI079	Poland	Obłazowa cave (WE)	III	14,376	mandible	194,416	7,570	1,631	8.72 (5.58)	92.09	8,426	0.24	0.27
MI080	Poland	Obłazowa cave (WE)	III	14,376	molar	1,130,490	121,210	11,358	64.50 (28.25)	98.21	9,090	0.26	0.25
MI081	Poland	Obłazowa cave (WE)	III	14,376	mandible	2,398,760	135,277	8,597	47.04 (20.01)	98.73	9,090	0.23	0.21
MI093	Poland	Nad Tunelem Cave	yellow	11,170	mandible	2,229,703	117,096	3,067	16.74 (9.01)	97.30	9,080	0.24	0.22
MI1105	Poland	Komarowa cave	B	14,180	molar	890,838	30,440	4,414	25.24 (12.03)	98.34	9,090	0.22	0.23
MI283	Slovakia	Peskö	4a	11,263	molar	1,053,800	231,909	2,066	10.95 (5.29)	97.60	9,028	0.21	0.22
MI361	Czech R.	Býčí skala	D8c	11,800	molar	501,882	16,027	7,467	41.59 (28.78)	97.38	9,031	0.16	0.15
MI362	Czech R.	Býčí skala	D8c	11,800	molar	357,243	35,050	8,483	47.02 (24.15)	98.60	9,090	0.23	0.23
Mg1	Russia	Southern Ural	—	0	tissue	634,069	—	—	—	100	9,090	—	—
Mg2	Russia	Southern Ural	—	0	tissue	330,820	—	—	—	100	9,090	—	—

*see Materials and Methods section for details.

^**^The fraction of DNA molecules with deaminated nucleotides at the terminal bases.

**Figure 2:**
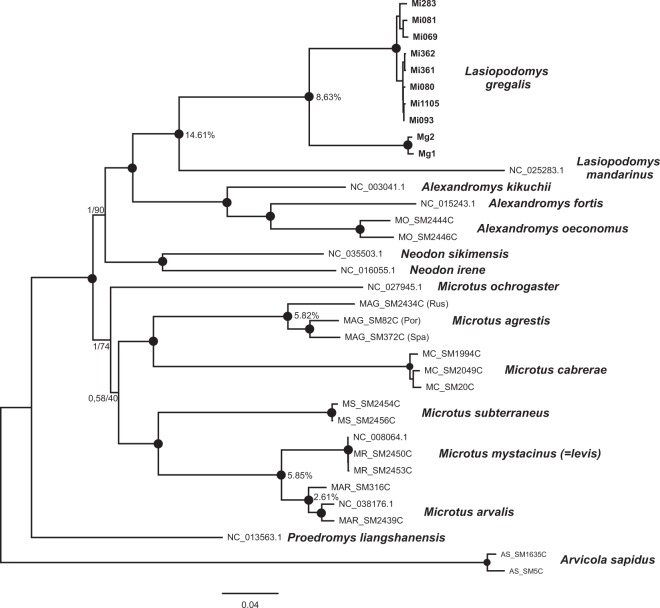
Bayesian phylogeny of Arvicolini based on the 9,090 bp fragment of mtDNA. The samples obtained in this study are written in bold. Nodes with posterior probabilities above 0.95 and bootstrap supports above 95 are marked with black dots. Otherwise posterior/bootstraps are displayed on the node. The percent of pairwise divergence between two branches is presented in front of the selected nodes (see text for details).

Using 9.09 kb of mtDNA, we reconstructed a phylogeny of all species from the *Microtus*, *Neodon*, *Alexandromys* and *Lasiopodomys* genera with available mtDNA genome sequences (Fig. 2). The resulting tree in general reassembled phylogenetic relationships previously inferred from mtDNA cytochrome *b* sequences^23^. The recovered tree consisted of two main branches, the first of which comprised the subgenera *Microtus* (*Microtus arvalis, Microtus mystacinus* (= *levis*)) and *Terricola* (*Microtus subterraneus*), which formed a sister clade to *Agricola* (*Microtus agrestis*) and *Iberomys* (*Microtus cabrerae*) with *Microtus ochrogaster* being the basal species. The second branch consisted of narrow-headed voles (*L. gregalis*) from both the extant and the Late Pleistocene, which clustered with *L. mandarinus* forming together a sister clade to the genus *Alexandromys* represented here by *A. fortis*, *A. oeconomus* and *A.kikuchii*. All the above-mentioned species were in a sister relationship to the Asiatic *Neodon* lineage (*N. irene* and *N. sikimensis*). The deep divergence between present-day narrow-headed voles from Russia and Late Pleistocene European ones is noteworthy. The total divergence (D_xy_) between these two groups was 8.6%, higher than between the Northern and both the Portuguese and the Mediterranean lineages of *Microtus agrestis* (5.89% and 5.72%, respectively), as well as between *Microtus arvalis* and *Microtus mystacinus* (=*levis*) (5.82%) (Fig. 2).

**Figure 3:**
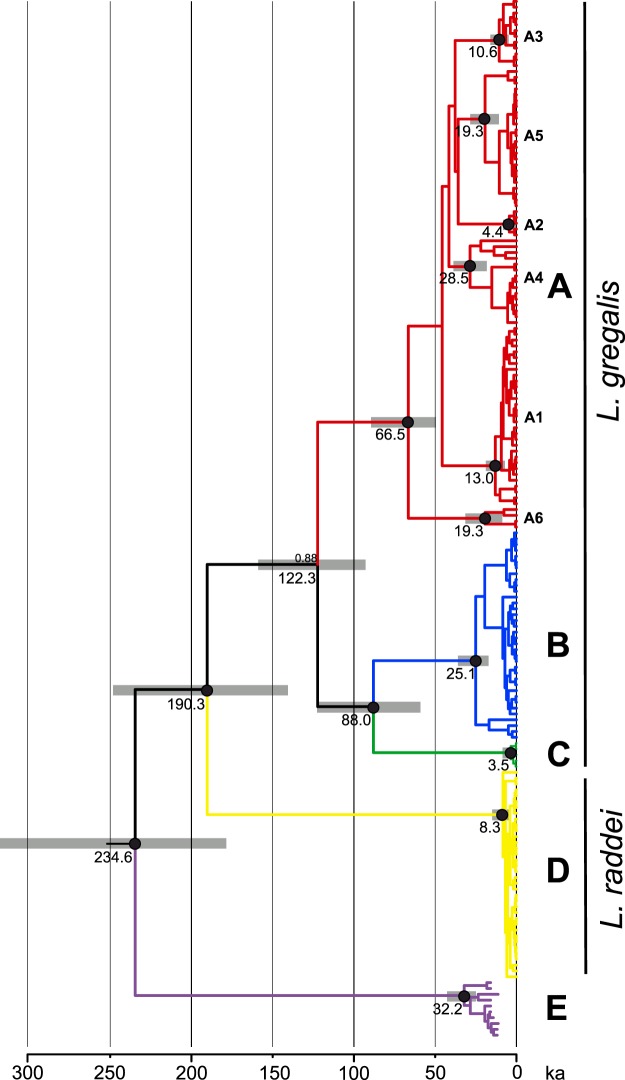
Bayesian phylogeny of extant and the Late Pleistocene narrow-headed voles based on the 889 bp fragment of mtDNA cytochrome *b*. The posterior probabilities of main nodes higher than 0.95 are indicated by black dots. Numbers below nodes are the estimated tMRCAs and the grey bars represent the 95% highest posterior density intervals of node ages. Node colours corresponds to those on Fig. 1.

To better characterise the relationships between the Late Pleistocene European and extant Asiatic narrow headed-vole populations we reconstructed the phylogeny based on mtDNA cytochrome *b* sequences of 179 specimens. It revealed five well supported lineages (Fig. 3). Four of them correspond to the previously described lineages A – D^16,17^, whereas the fifth, designated as E, was basal with respect to all others and included the narrow-headed voles from Europe. The divergence between lineage E and the others ranged between 8.8% (E-A) and 10.5% (E-B), which was slightly lower than the divergence between lineage D and the others (range: 10.4% (D-A) −11.5% (D-B)).

The phylogeny was calibrated using the substitution rate of 4 × 10^−7^ substitutions/site/year^−1^, without additional fossil calibration. This resulted in much younger divergence estimates of mtDNA lineages than previously reported^16^. Most of the genetic diversity of the extant Asiatic populations coalesced between 15 and 10 ka. Within lineage A, sublineage divergence was estimated as ranging between 65 and 35 ka. The divergence of lineages A, B and C was estimated as ranging between 125 and 90 ka. The divergence between lineage D, which was recently established as *Lasiopodomys raddei* and lineages A-C was estimated as ca. 190 ka, whereas the divergence of newly recognised lineage E was estimated as ca. 230 ka (Fig. 3).

To include in the analysis sequences of the 21 Late Pleistocene narrow-headed voles from Urals^18^ we reconstructed also the phylogeny based on a shorter fragment of mtDNA cytochrome *b* (461 bp). The resulting tree had the same topology as the one reconstructed from the longer fragment, only the divergence times of the main lineages were slightly older (Fig. S1).

## Discussion and Conclusions

For many years, the narrow-headed vole was included in the genus *Microtus* within the separate subgenus *Stenocranius*, based on skull shape characteristics^24,25^. From the 1980s, far before the application of molecular markers to vole systematics and phylogeny, palaeontologists concluded that the narrow-headed vole is one of the earliest evolutionary offshoots of rootless voles derived from the *Allophaiomys* stock. As a consequence, *Stenocranius* was thereafter considered to represent a distinct phylogenetic lineage of *Microtus* s. l.^26,27^. It is represented by an ancestor-descendant array of species „*hintoni*” – „*gregaloides*”– „*gregalis*” first recognised by Fejfar and Horáček^28^. However, this point of view was not widely accepted until the 1990s, when the morphological feature of the first lower molar called “*Pitymys*-like rhombus”, for which the early forms of the clade were traditionally regarded as “*Pitymys hintoni*” and “*Pitymys gregaloides*”^4,20^, appeared merely as a common ancestral feature in several genera lineages, rather than a prove of their taxonomic relation^26,29,30^.

Mezhzherin et al.^31^ were perhaps the first to reveal a close relationship between *Microtus gregalis* and *Lasiopodomys brandtii* (together with *A. oeconomus*, *A. middendorffii*, and *A. fortis)* based on allozyme studies. The first application of molecular tools to explore species limits in *Microtus*, especially inference based on mtDNA cytochrome *b* analysis^32^ did not clarify the taxonomic status of the narrow-headed vole. The species *M. gregalis* was the most divergent *Microtus* species, occupying an unstable phylogenetic position. Results from further studies performed in recent years, in particular the evidence gathered from nuclear genes^33,34^, have supported the sister relationship of *Stenocranius* with representatives of the *Lasiopodomys* (*L. mandarinus* and *L. brandtii*) genus. Its taxonomic status within *Lasiopodomys* is currently accepted by taxonomists^35^. Our phylogeny, based on a large portion of mtDNA genome, is consistent with those findings. The position of *L. gregalis* together with *L. mandarinus* is well supported by both used phylogenetic methods. However, the *Lasiopodomys* clade is not differentiated from all other *Microtus* groups but is in a sister relationship with the Asiatic lineages *Alexandromys* and *Neodon* as suggested from previous allozyme^31^ and mtDNA cytochrome *b* studies^23^.

MtDNA cytochrome *b*-based phylogeny provided a more accurate characterisation of the relationship between European and Asiatic narrow-headed voles, and confirmed a high genetic divergence between them. However, the estimated divergence times of Asiatic lineages were much younger than in the previous study^16^ where the phylogeny was calibrated using combined heterochronous genetic and fossil data. It was suggested that due to the time-dependence of molecular rates, fossil calibration might overestimate the timing of intraspecific events^36^. The substitution rates of mtDNA cytochrome *b* were recently estimated for a range of small mammal species using either heterochronous data or biogeographic events. All estimations yielded similar mutation rates: 3.27 × 10^−7^ (substitutions/site/year^−1^) for *Microtus arvalis*^37^, 3.88 × 10^−7^ and 4.57 × 10^−7^ for *Microtus agrestis*^38,39^, 1.53 × 10^**−7**^ and 4.63 × 10^−7^ for *Dicrostonyx torquatus*^40,41^ and 5.38 × 10^−7^ for *Ctenomys sociabilis*^41,42^. It was shown that, across mammals, mtDNA mutation rates correlate with several traits such as lifespan, body mass and generation turnover rate^43^. Horn *et al*.^44^ found a correlation between rodents’ rates and lifespan. Therefore, an mtDNA cytochrome *b* substitution rate around 4 × 10^−7^ substitutions/nucleotide/year^−1^ used here is possibly universal for the *Microtus* genus and other rodents with similar life history. Interestingly, most of the genetic diversity of the extant Asiatic populations coalesce between 15 and 10 ka (Fig. 3), suggesting that it arose mostly during the Late Glacial and the Holocene, and that climate changes at the end of the Pleistocene and the Pleistocene to Holocene transition might have exerted a larger impact on narrow-headed vole populations than previously thought.

The divergence of lineage E, which includes all European specimens, was estimated at around 230 ka. The used substitution rates may be applied to estimate the divergence of younger branches of the narrow-headed voles’ tree, although it is not clear whether they are still valid to estimate the age of highly divergent lineages, such as D and E. Although, “intraspecific” substitution rates were recently used to estimate the ages of highly divergent lineages and species within the *M. agrestis* and *M. arvalis* species groups^45^, this estimation should be interpreted with caution and regarded as minimal. *Lasiopodomys gregalis* appeared for the first time in the European fossil record in the Middle Pleistocene, probably in MIS 17, an equivalent of Cromerian Interglacial II^27^, or in MIS 15, correlated with Muchkap Stage Interglacial^46^, ca. 0.7–0.5 Ma. This may be regarded as the maximal estimate of lineage E divergence.

Both phylogenetic reconstructions clearly showed that European post-LGM narrow-headed voles represent a highly divergent lineage from contemporary and the Late Pleistocene Asiatic ones. This new evolutionary unit does not match those of any currently recognised haplogroups of extant species of *Lasiopodomys*, such as *L*. *gregalis*^16^ or *L. raddei*^17^. The observed divergence from the extant Asiatic populations (8.8–10.4%) is higher than between the lineages within the *Microtus agrestis* (3–6%)^45^ or *Microtus arvalis* (4–9%) groups^47^, currently considered by most specialists as a separate species or close to a separate species^35^.

Interestingly, the results of Ecological Niche Modelling showed little or no suitable narrow-headed voles’ habitats in Central and Western Europe during the LGM^18^. It was proposed that Late Pleistocene European populations may have had a broader or different ecological niche than the Asiatic ones, possibly as a consequence of local adaptations^18^. This suggests that the divergence of the European narrow-headed voles might have been a consequence of habitat fragmentation and isolation of the European population in the refugial area during one of the interglacials, possibly Holsteinian (MIS 11). This could have led to the rapid evolutionary change and shift of the ecological niche of the narrow-headed voles. Similar mechanism was recently suggested for the extant Norwegian lemmings which survived the LGM in the isolated refugium in Scandinavia^48^.

Given the lack of clear morphological differentiation, possible different ecological niche and especially the phylogenetic placement of European narrow-headed voles as a sister taxon against *L. gregalis* and *L. raddei*, we suggest that it should be considered as a cryptic species within the *Lasopodomys gregalis* species group.

Species designations follow nomenclatural priorities, which also apply to genetically distinct lineages. In Europe, the Late Pleistocene fossil narrow-headed vole was first recognised by Nehring (1875)^49^ who found “*Arvicola gregalis*” in Thiede near Wolfenbuttel, Central Germany. Woldřich^50^ reported this species from rich fossil assemblages in Sudslavice, South Bohemia, and later based on multiple records from Moravian and Austrian caves’ deposits, identifying it as an index fossil of glacial communities^50^. Newton^51^, simultaneously described this species as *Microtus* (=*Arvicola*) *gregalis* based on specimens from Ightham Fissures, Kent, England. This species was later described by Hinton^52^ as a new one - *Microtus anglicus*. Among the numerous collections from Ightham Fissures, Hinton^52^ designated the type specimen as a nearly complete adult skull, essentially similar to the extant representatives of *Stenocranius*. In the same description he also indicated a greatly reduced fourth outer angle in the first lower molar. This was the basis on which *Microtus anglicus* was later synonymised with *Microtus gregalis* by most authors^4,20,28^. Thus, in terms of the taxonomic nomenclature the name *Lasiopodomys anglicus* has priority in designation of Late Pleistocene narrow-headed vole lineage from Europe as a species.

Among the presented results, the most interesting aspect is their incompatibility with the available paleobiogeographic hypotheses. The traditional view on the history of the European narrow-headed voles predicts its complete disappearance during the interglacials, with woodland vegetation, and mass re-expansion during glacial stages from the core distribution area beyond the limits of Europe. Its distribution throughout the Weichselian fits that hypothesis quite well, as the species first appears as a rare element by the end of MIS 4, becoming a dominant form only during MIS 3^12,21,53,54^. The present results suggest no occurrence of distant migrations, requiring the species to survive interglacial stages in refugia within Europe (supposedly in the alpine mountains or in Scandinavia). However, to our knowledge, no reliable records are available that support this hypothesis. *Lasiopodomys anglicus* has been an index species and the dominant component of glacial communities throughout the Middle and Late Pleistocene of Central Europe. The uncertainties on its paleobiogeography’s real dynamics indicate that our knowledge of the Quaternary past remains incomplete, requiring urgent further research.

## Materials and Methods

### Samples

Narrow-headed vole remains were obtained from palaeontological sites in Poland (Obłazowa cave WE, Nad Tunelem cave, Komarowa cave), Czech Republic (Býčí Skala cave) and Slovakia (Peskö cave). The samples were collected from layers dated to the Latest Pleistocene, between 20 and 11 ka: layer III from Obłazowa WE, layer B from Komarowa, layer 8a from Býčí Skala and layer 4 from Peskö^55–57^ (Fig. 1). Four samples from Nad Tunelem cave were radiocarbon dated in the Poznań Radiocarbon Laboratory to determine the layer’s age (Supplementary Table S1). Radiocarbon ages were calibrated with Oxcal 4.2^58^ using IntCal13 calibration curve^59^. Ethanol-preserved tissue fragments of two present-day narrow-headed voles were obtained from specimens caught in Ural Mountains.

### DNA extraction, enrichment and sequencing

Prior to laboratory procedures, all samples were photographically documented. All experimental procedures were performed in a laboratory dedicated to ancient DNA work in the Laboratory of Paleogenetics and Conservation Genetics, Centre of New Technologies, the University of Warsaw. Strict precautions were taken to avoid contamination. Teeth were thoroughly rinsed with water, submerged in extraction buffer (0.5 M EDTA pH = 8.0; 0.5% N-Laurylsarcosine; 0.1 mg Proteinase K) and crushed with a pipette tip. DNA was extracted according to a protocol optimised for retrieval of short DNA molecules^60^. After overnight incubation, one part of extraction buffer was mixed with thirteen parts of binding buffer (5 M guanidine hydrochloride, 40% isopropanol) and eluted through MinElute silica column (Qiagen). Silica suspension was washed twice with 750 µl of PE buffer (Qiagen) and DNA was eluted twice from columns, each time using 30 µl of pre-warmed EB buffer. 20 µl of DNA extracts were converted into double-stranded, double-indexed sequencing libraries according to a previous protocol^61^ with the following modifications: after blunt-end repair, instead of undergoing SPRI clean-up, enzymes were heat-inactivated for 20 min at 75 °C. In the ligation step, sample cross-talk during sequencing was minimised using P5 and P7 adaptors with 7 bp barcodes at the ends, in addition to standard Illumina indexes, as previously described^62^. Final indexing PCR was performed in three parallel reactions using 19 cycles and AmpliTaqGold MasterMix (Thermo Fisher Scientific). Sequencing libraries were enriched for *L. gregalis* mtDNA, as previously described^63^. Hybridisation DNA bait was produced using ethanol-preserved muscle fragments of two present-day *L. gregalis* specimens. DNA was extracted using the DNeasy Blood & Tissue Kit (Qiagen) and mtDNA was amplified as a set of different length amplicons designed on the *M. arvalis* (NC_038176.1) and *M. mystacinus* (=*levis*) (NC_008064.1) mtDNA genome sequences. PCR products were mixed in equimolar ratios and sheared to the length of ca. 200 bp using the Covaris S220 sonicator. Shared DNA was converted into bait as previously described^63^. Two hybridisation runs were performed on library pools from up to five specimens. Enriched libraries were amplified for 15 cycles after each round, quantified with qPCR (Illumina Library Quantification kit, KAPA), pooled and sequenced with other libraries on the NextSeq platform using the 2 × 75 bp paired end mode. Additionally, 10 µl of shared mtDNA of modern *L. gregalis* were also transformed into sequencing libraries as described above but with 12 cycles of indexing PCR, after which they were sequenced.

Sequencing reads were demultiplexed using Bcl2fastq. Reads containing the appropriate barcode were filtered with Sabre script, after which the AdapterRemoval v.2^64^ was used to collapse overlapping reads. mtDNA genome sequences of two present-day *L. gregalis* were assembled *de novo* using NOVOPlasty^65^ and annotated using MITOS^66^. The resulting mtDNA sequence was used as a reference to map the reads from Late Pleistocene samples. Mapping was performed using the mem algorithm in *bwa* with option -B 1 to allow mapping of divergent reads. Duplicates were removed; variants and consensus sequences were called using samtools and bcftools^67^. Only reads with mapping quality over 30 and longer than 30 bp were retained and only positions with minimum 2x coverage were called. Each bam alignment was manually inspected using Tablet^68^. The presence of excessive DNA damage at the molecules’ ends was checked with mapDamage v.2^69^.

### Phylogenetic analyses

Phylogenetic analyses were performed on two datasets; the phylogenetic position of European narrow-headed voles within *Arvicolini* was investigated using the dataset comprising mtDNA genome sequences of 15 species from genus *Microtus*, *Lasiopodomys* and *Neodon*, together with sequences of two extant and ten Late Pleistocene *L. gregalis* obtained in this study (Table 1). To include data pertaining to several recently published species, the alignment length was limited to 9.09 kb of mtDNA.

Phylogeny was reconstructed using the Maximum Likelihood (ML) and Bayesian approaches. For both analysis the partitioning scheme and substitution model were chosen using PartitionFinder2^70^ (Supplementary Table S2). The ML tree was reconstructed using RaxML v.8^71^, the best tree was selected from 20 ML searches, and the support was assessed with 100 rapid bootstraps. The Bayesian tree was reconstructed using ExaBayes 1.5^72^. Two runs, each with four coupled chains were run for two million generations sampled every 500 generations. Default values were used for tuning and branch swap parameters. Convergence and sampling were assessed in Tracer 1.7^73^. For all parameters, the EES values were above 200.

The relationships between European and Asiatic populations of narrow-headed voles were investigated using a dataset comprising 179 previously published partial cytochrome *b* mtDNA sequences (889 bp) of *Lasiopodomys gregalis* (n = 28; KF751077–KF751104), (n = 133; KJ192239–KJ192327), (n = 2; AF163895, AY513803), (n = 2; GQ352466- GQ352467), (n = 1; AF429817), as well as those obtained in the present study.

Bayesian phylogeny was reconstructed using the Beast 1.8.4^74^ and the best fitting substitution model was selected using jModeltest-2. The phylogeny was calibrated using a substitution rate of 4 × 10^−7^ substitutions/site/year^−1^. Late Pleistocene samples were dated by stratigraphic layers, the ages of which was estimated using ^14^C dating. Each sample was assigned an age corresponding to the mean form the age range formed by the youngest and oldest values of the 95.4% probability distributions of all calibrated radiocarbon dates known for the layer (Table 1). Marginal Likelihood Estimation (MLE) was used to test which clock model and tree prior best fitted the data^75^. The constant population sizes were compared to SkyGrid tree priors^76^ and strict versus relaxed lognormal clock models. For each tree prior/clock model combination analysis was run for 100 million generations with trees sampled every 10,000 generations. MLEs were estimated using path sampling and stepping stone sampling analyses, both with 1,000 steps run for 100,000 generations sampled every 1,000 generations. Bayes factors strongly favoured the SkyGrid tree prior combined with the relaxed lognormal clock model over all other combinations (logBF > 10). The selected model was used to run additional analysis using the same MCMC settings. The trees from two runs were combined in *logcombiner* and the Maximum Clade Credibility tree was selected from among the 9,000 trees using the *treeannotator*.

To include in the analysis also the Late Pleistocene and Holocene (n = 32; KC295791- KC295822) narrow-headed voles from the Ural Mountains published earlier^18^ we also reconstructed phylogeny based on 461 bp mtDNA cytb fragment. The phylogeny was reconstructed in Beast 1.8.4 using identical settings as for the longer fragment.

The genetic divergence (Dxy) between sequence groups was estimated in DnaSP v6^77^.

## Supplementary material

Supplementary Information

## Data Availability

Sequences obtained in this study was deposited in GenBank under accession no. MN199169 – MN199180.
