# Supplementary material for: Highly divergent lineage of narrow-headed vole from the Late Pleistocene Europe
Source: Sci Rep. 2019 Nov 28;9:17799. doi: 10.1038/s41598-019-53937-1 (PMC6882798; doi:10.1038/s41598-019-53937-1)
Supplement: Supplementary file 1 — Supplementary Information [file 41598_2019_53937_MOESM1_ESM.pdf]

SUPPLEMENTARY INFORMATION

Highly divergent lineage of narrow-headed vole from the Late Pleistocene Europe

Mateusz Baca[1], Danijela Popović[1], Anna Lemanik[2], Katarzyna Baca[1], Ivan Horáček[3], Adam Nadachowski[2,*]

[1] - Centre of New Technologies, University of Warsaw, Banacha 2c, 02-097 Warsaw, Poland

[2] - Institute of Systematics and Evolution of Animals, Polish Academy of Sciences, Sławkowska 17, 31-016 Krakow, Poland

[3] - Department of Zoology, Charles University, Viničná 7, 128 44 Prague, Czech Republic

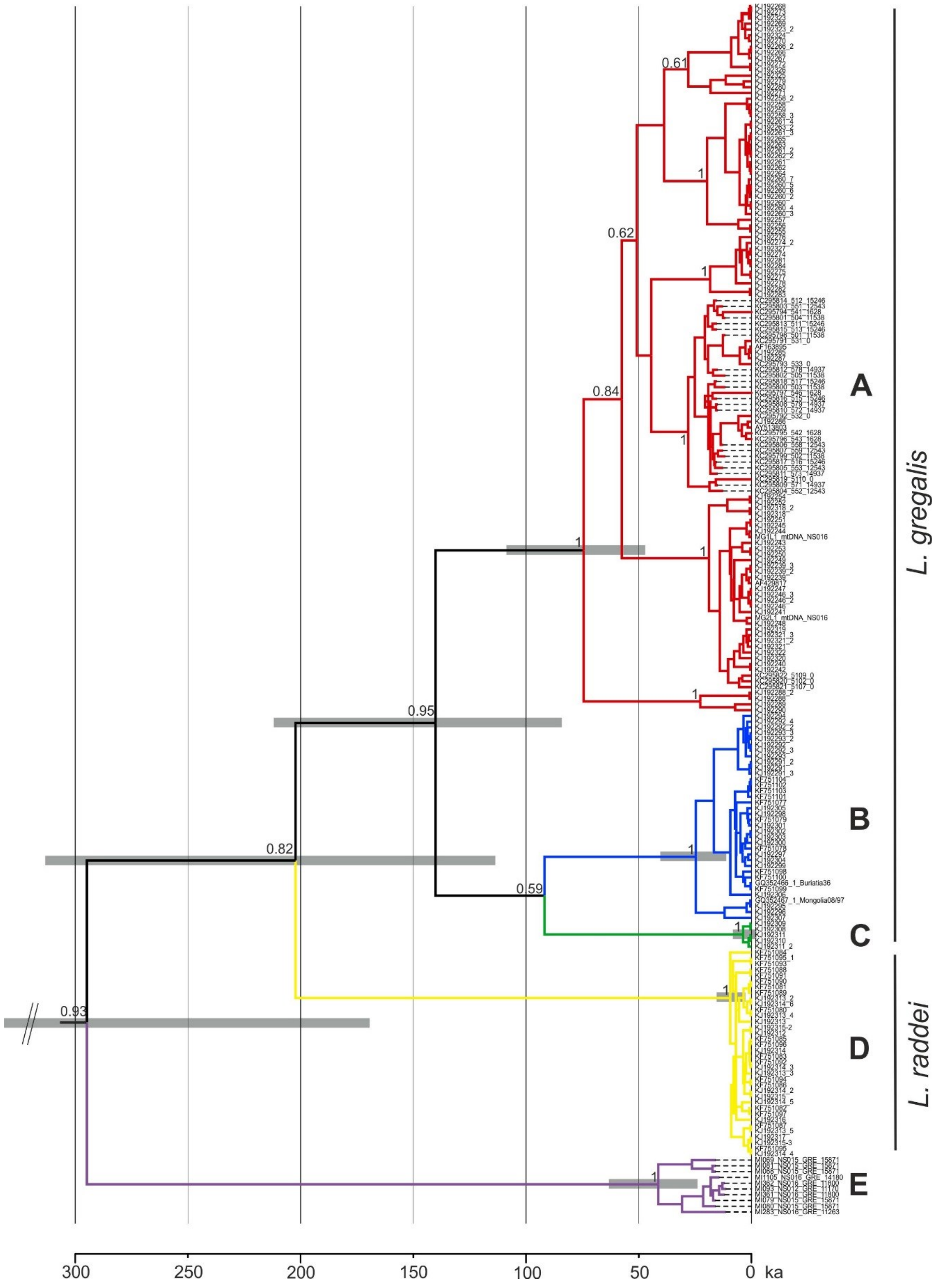

Figure S1. Bayesian phylogeny of narrow-headed voles.

Maximum Clade Credibility tree of the Late Pleistocene and extant narrow-headed voles based on 461 bp fragment of mtDNA cytochrome b. Number above nodes denotes posterior probability of branches. Grey bars represent 95% highest posterior density of node ages. Colours of the nodes correspond to those on Figure 1.

Table S1. Radiocarbon dates obtained in this study.

Sample	Country	Site	Layer	Lab ID	^{14}C date	Calibrated age (cal BP)			C% coll	N% coll	C/N coll
						lower	upper	median			
MI107	Poland	Nad Tunelem cave	yellow	Poz-90385	9740 ± 60 BP	11262	10813	11173	42.1	14.8	3.3
MI108	Poland	Nad Tunelem cave	yellow	Poz-90387	9730 ± 60 BP	11252	10801	11161	44.7	15.8	3.3
MI109	Poland	Nad Tunelem cave	gray	Poz-90388	10030 ± 60 BP	11802	11271	11534	44.7	16.0	3.3
MI110	Poland	Nad Tunelem cave	gray	Poz-90389	9670 ± 50 BP	11211	10789	11094	42.9	15.1	3.3

Table S2. Partitioning scheme and substitution models used in the phylogeny reconstruction.

No	Model	sites	data blocks	positions
1	GTR+I+G+X	291	ATP8_1, ATP8_2, ND2_1, NAD6_2	2854-3014\3 2855-3014\3 1-27\3 7352-7871\3
2	GTR+I+G+X	1360	ATP6_2, ND2_2, cox1_2, cox2_2, cytb_2	3016-3694\3 2-27\3 419-1962\3 2103-2785\3 7949-9090\3
3	GTR+I+G+X	1292	ATP6_3, cox3_3, ND2_3, ATP8_3, cox1_3, cox2_3	3017-3694\3 3697-4478\3 3-27\3 2856-3014\3 420-1962\3 2104-2785\3
4	GTR+I+G+X	729	NAD5_1, non, trnH	5539-7350\3 28-29 97-97 173-174 245-281 417-417 2029-2032 2101-2101 2786-2788 7942-7947 5341-5408
5	GTR+I+G+X	2127	cox2_1, cox1_1, ATP6_1, NAD4_2, trnW, trnG, trnS1, trnK, cox3_1, cytb_1	2102-2785\3 418-1962\3 3015-3694\3 4530-5340\3 30-96 4479-4528 5409-5469 2789-2853 3695-4478\3 7948-9090\3
6	GTR+I+G+X	1862	NAD4_3, NAD5_2, cox3_2, trnC, trnY, treS2, trnA, trnL1, NAD6_1, trnD, trnE, trnN	4531-5340\3 5540-7350\3 3696-4478\3 282-349 350-416 1963-2028 98-172 5470-5538 7351-7871\3 2033-2100 7872-7941 175-244
7	GTR+I+G+X	1429	NAD4_1, NAD5_3, cytb_3, NAD6_3	4529-5340\3 5541-7350\3 7950-9090\3 7353-7871\3
